# Xylazine-Induced Skin Ulcers in a Person Who Injects Drugs in Philadelphia, Pennsylvania, USA

**DOI:** 10.7759/cureus.28160

**Published:** 2022-08-19

**Authors:** Srikrishna V Malayala, Bhavani Nagendra Papudesi, Raymond Bobb, Aliya Wimbush

**Affiliations:** 1 Addiction Medicine, Merakey, Philadelphia, USA; 2 Internal Medicine, Suburban Hospital, Philadelphia, USA

**Keywords:** substance use disorder (sud), intravenous drug user, foot ulcer, chronic ulcer, drug withdrawal, herione withdrawal, opiate use, opioid withdrawal, drug addiction, xyalazine

## Abstract

Xylazine, an alpha-2 adrenergic receptor agonist typically used as a sedative and analgesic in veterinary medicine, is being illicitly supplied to persons who inject drugs (PWID), especially in Puerto Rico and Philadelphia, Pennsylvania in the USA. There is a high prevalence (up to 78%) of xylazine in fentanyl in these areas and also a steep increase in fatalities from its overdose.

In this case report, we discuss a case of xylazine-induced skin ulcers in a PWID in the city of Philadelphia. The patient is a 37-year-old female who was injecting about eight to ten "bags" of "dope" (fentanyl, which is typically mixed with xylazine in Philadelphia) every day. She typically injected into her veins on the hands and sometimes into the legs. She presented with ulcers on her lower extremities extending from the knees to ankles, associated with copious purulent drainage and a foul smell. There was extensive necrosis of the subcutaneous tissues, abscesses, and tibial osteomyelitis. This led to multiple hospitalizations with bacteremia from Strep pyogenes, methicillin-resistant *Staphylococcus aureus*, methicillin-sensitive *S. aureus*, *Enterococcus faecalis*, *Escherichia coli*, and Proteus requiring intravenous antibiotics. She required debridement of the wounds and topical care to treat them.

In the areas with a high prevalence of the use of xylazine mixed with fentanyl or heroin, abscesses, and painful skin ulcers are very often reported. The mechanism is thought to be due to its direct vasoconstricting effect on local blood vessels and the resultant decreased skin perfusion. Prolonged use can lead to decreased perfusion and impaired wound healing, leading to higher chances of infection of these ulcers. In addition to the topical effect of vasoconstriction, xylazine also leads to hypotension, bradycardia, and respiratory depression.

A skin ulcer in a PWID, similar to the ones reported in our case, should raise clinical suspicion for the presence of xylazine in opiates and other substances.

## Introduction

Xylazine is an alpha-2 adrenergic receptor agonist, similar to clonidine, and is a non-narcotic sedative used for analgesia and muscle relaxation exclusively in veterinary medicine [1]. Illicit use of xylazine among persons who inject drugs (PWID) has been reported in Puerto Rico since the early 2000s [2] and more recently in Philadelphia, Pennsylvania [3]​​. As of now, there is no precise categorization or confirmatory evidence regarding the trends, geographical distribution, and health risks.

In veterinary medicine, xylazine has been termed as 'anestesia de caballo' (horse anesthetic) [4]. In Philadelphia, xylazine has been more popularly termed as 'tranq', and the more commonly used illicit drugs, heroin and fentanyl cut with xylazine, have been referred to as 'tranq dope' [3]. In the United States, Xylazine is not a scheduled medication. Although it is approved for veterinary medicine, the Food and Drug Administration has not approved it for human use.

From 2000 to 2006, cocaine was the leading drug associated with overdose deaths, which was replaced successively by prescription opioids (2007-2013), heroin (2014-2015), and illicitly-manufactured fentanyl (2016-present) [5]. In more recent years, a sharp increase in fatalities has been linked to systemic polysubstance use and potent synthetic compounds in numerous drug classes, including synthetic opioids such as fentanyl [6], sedatives like benzodiazepines, and stimulants such as methamphetamine [7], and novel benzodiazepines [8]. Recent news articles report a high prevalence (up to 78%) of xylazine in fentanyl screen-positive urine samples in Puerto Rico and Philadelphia and also a steep increase in fatalities from xylazine overdose [9]. In a recent report from Philadelphia, xylazine increased from being detected in less than 2% of cases of fatal heroin or fentanyl overdose between 2010 and 2015 to 31% of fatal heroin or fentanyl overdose cases in 2019 [10].

With this extent of the use of xylazine and its malicious effects, there is an urgent necessity to focus on the manifestations of xylazine on PWID, the dynamics of its usage, and fatality trends to understand its role in shifting the current US overdose dynamics. In this case report, we present a case of skin ulcers that were noticed in a PWID in the city of Philadelphia. We also hypothesize the presumed mechanism of skin injury and illustrate the latest trends in xylazine usage, its morbidity, and mortality trends all across the USA. We intend to increase suspicion of novel health risks of xylazine, specifically skin ulcers and abscesses.

## Case presentation

The patient in this case report is a 37-year-old Caucasian female with a known history of opiate use disorder. She had a very long history of polysubstance abuse and injection drug use. She was homeless at that time, living off the streets. She is a resident of Philadelphia City. She was on a methadone maintenance program (MMT), taking 80 mg of methadone daily but on an irregular basis. Apart from drug use, she also had a history of multiple infectious complications from drug use, including left clavicle osteomyelitis, left sternoclavicular septic arthritis, and multiple bacteremias. Most of her treatments were incomplete in the past as she had a tendency to leave the hospital against medical advice (AMA) in the middle of her hospitalizations. She also had a prolonged QT interval at the baseline, measuring approximately 480 ms, likely due to the concurrent use of methadone and illicit opiates.

**Figure 1:**
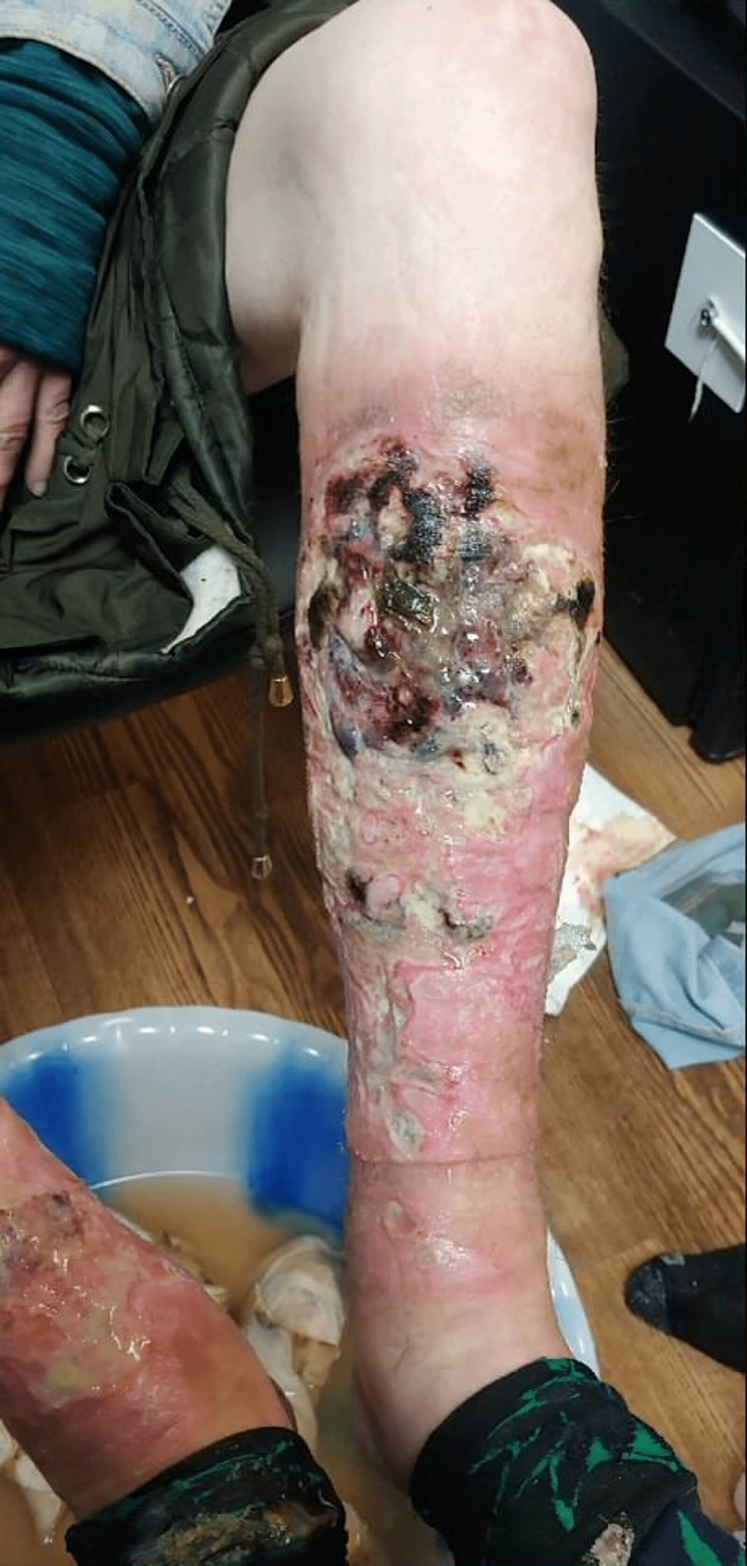
Xylazine-induced leg skin ulcers, cellulitis and osteomyelitis in a person who injects drugs in Philadelphia

**Figure 2:**
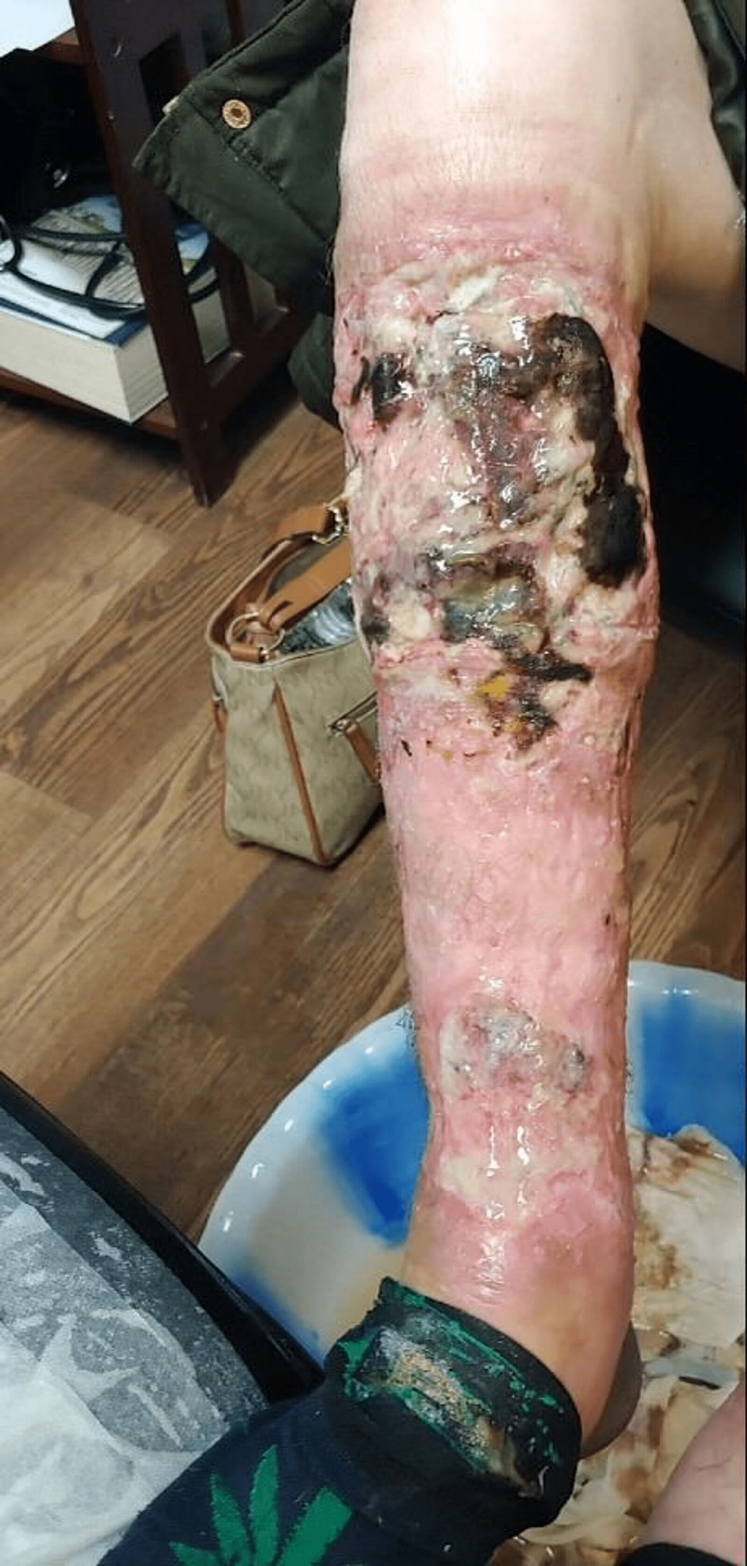
Xylazine-induced right leg cellulitis, wound infection and osteomyelitis in a person who injects drugs

She presented to the addiction medicine office for a follow-up visit. During that time, she was using about 10 bags of intravenous fentanyl/heroin every day and around five alprazolam "bars" every day. She was also smoking a pack of cigarettes every day. At this clinic visit, she reported ulcers on her lower extremities (Figures 1-2) that started a few weeks ago and gradually got worse. She had a low-grade fever of 99.8 °F, a blood pressure of 100/48 mm Hg, and a heart rate of 92/minute. The ulcers were foul-smelling, had copious purulent drainage, and were present on both the anterior aspects of her legs, extending from just below the knee to above the ankle. They were extending into the bone at some places.

She reported that the ulcers started spontaneously a few weeks ago and gradually got worse. She denied any injury or insect bites that might have led to the wounds. She usually injects into her hands, but sometimes into her legs as well. Whenever she missed a vein, she noticed that the areas were ulcerated. She reported that these wounds were progressively getting worse, unlike any of her prior wounds.

After addressing the dose of methadone, she was advised to go to the emergency room for the management of the ulcers. In the emergency room, she had a CT scan of her lower extremities that showed acute and active osteomyelitis in the proximal third of the right anterior and anterolateral tibia. There was also a soft tissue abscess in the right anterior compartment musculature of the proximal third of the leg and one more abscess in the right anteromedial soft tissues of the proximal and mid-leg region. On the left leg, there was no cortical irregularity to suggest osteomyelitis of the left lower extremity, but the findings were consistent with a soft tissue abscess and cellulitis. She was admitted to the hospital with broad-spectrum antibiotics including intravenous Vancomycin and Piperacillin/Tazobactam. Subsequently, when the patient refused to get any blood draws to monitor their therapeutic vancomycin level, the antibiotic was changed to intravenous daptomycin. The lower extremity wounds were debrided at the bedside. Topical wound care was offered and continued. Pain management and opiate withdrawal management were continued without any interruptions. The methadone dose was titrated while she was admitted. However, she left against medical advice from the hospital, and her ongoing injection opiate use continued. The blood cultures in this hospitalization grew Streptococcus pyogenes.

She presented to the emergency room and was admitted five times over the next eight weeks with very similar presentations, and at each visit, she was admitted with the resumption of intravenous antibiotics and topical wound care. She had bacteremia from methicillin-resistant *Staphylococcus aureus*, *Proteus mirabilis*, methicillin-sensitive *S. aureus*, and Enterobacter species. She was also evaluated by a plastic surgeon who recommended definitive reconstruction of the wounds once the infection resolves. In the last hospitalization, the patient stayed in the hospital for a few weeks and completed the recommended treatment. She was discharged to a recovery house with oral antibiotics (levofloxacin and doxycycline) for eight more weeks. She was advised to apply Silvadene ointment to the wounds, wash them with an antiseptic solution, and redress them with Xeroform, ABD pads, and Kerlix.

## Discussion

Addiction is one of the most significant hardships and one of the most challenging clinical conditions to deal with, especially without external support or interventions. The consequences are much worse when they cause new medical conditions or worsening of comorbid conditions and pre-existing diseases in a patient who is already apprehensive about seeking healthcare due to multiple factors like the fear of being judged, social stigma, poor economic status, poor health care awareness and limited access to healthcare. These patients typically tend to seek help or present to the hospital only at late stages, which makes our case report very pertinent.

Xylazine was discovered in 1962 in Leverkusen, Germany, and was used as an anti-hypertensive agent [11]. It is a partial alpha-2 adrenergic agonist. Peripherally, alpha-2 agonist causes arterial constriction, but centrally, it acts as a sympathetic antagonist, decreasing heart rate and contractility [11].

Xylazine can be swallowed, inhaled, smoked, snorted, or injected into the muscle or vein. There are no data on vaping. Intoxication mimics clonidine and tizanidine as they all share the same mechanism of action. The most common side effects in humans include transient hypertension secondary to vagus nerve stimulation, bradycardia, respiratory depression, hypotension, acidemia, coma, and a decrease in cardiac output [5]. Other rare but very concerning side effects that can occur are areflexia, asthenia, ataxia, blurred vision, disorientation, dizziness, drowsiness, dysarthria, dysmetria, fainting, hyporeflexia, slurred speech, somnolence, staggering, coma, apnea, shallow breathing, sleepiness, premature ventricular contraction, tachycardia, miosis, dry mouth, hyperglycemia, and diabetes [12]. It is also reported to cause hypotonia, dry mouth, urinary incontinence, and nonspecific electrocardiographic ST-segment changes [5]. Xylazine has a rapid onset within minutes and can last for eight hours or longer depending upon the dose, route of administration, and whether it was mixed with opioids or other drugs. But the duration of symptoms after an overdose can vary widely, all the way from 8 to 72 hours [5]. Due to all these side effects, it was not approved by the FDA for use in humans. It is to be noted that the traditional drug screen does not detect the presence of xylazine in urine or blood samples.

A high prevalence of abscesses and painful skin ulcers [13] developed over various body parts irrespective of the IV injection site was reported. The mechanism is thought to be mediated by its direct vasoconstricting effect on local blood vessels and resultant decreased skin perfusion [6]. In addition to vasoconstriction, it causes hypotension, bradycardia, and respiratory depression, leading to lower tissue oxygenation in the skin [14]. Thus, chronic use of xylazine can progress the vasoconstriction and skin oxygenation deficit, leading to severe soft tissue infections, including abscesses, cellulitis, and skin ulceration. Decreased perfusion also leads to impaired healing of wounds and a higher chance of infection of these ulcers [15].

In a recent study mentioning data from ten jurisdictions representing all four U.S. Census regions, xylazine was increasingly present in overdose deaths. The highest xylazine prevalence data was observed in Philadelphia (25.8% of deaths), followed by Maryland (19.3%) and Connecticut (10.2%). Illicitly manufactured fentanyl was present in 98.4% of xylazine present-overdose-deaths, suggesting a solid ecological link between fentanyl and xylazine compared to fentanyl’s association with other illicit substances such as cocaine (45.4%), benzodiazepines (28.4%), heroin (23.3%), and alcohol (19.7%). PWIDs in Philadelphia described Xylazine as a "sought after" adulterant that lengthens the short duration of fentanyl injection’s effects [7]. It is also used as a cutting agent for other opioid drugs. Some of the PWID who took xylazine without knowing may not report that they had it but ask the patient a few questions like "do you sometimes do not experience the usual dope like the high feeling after taking dope but feel excessively tired and dry mouth after having "dope"? This may lead the patient to enter a vicious cycle of using more illicit substances. One more important thing to consider from these studies on rodents is that the incidence and severity of corneal lesions are prevented or reduced with the administration of Yohimbine (an α-2 adrenergic antagonist) [15]. This could shed some light and lead to some clues in research on the treatment of cases of acute or chronic side effects of xylazine in PWID.

## Conclusions

There has been a rapid increase in the adulteration of illicit IV drugs with highly harmful substances, particularly those that are not even approved for human use. These substances are used for a multitude of reasons, like prolonging the duration of effect, using them as a cutting agent, and increasing the ease of availability of the base drug. The use of xylazine is rapidly becoming more frequent, and there is a potential risk that in the near future, this may be used alone or combined with a variety of illicit drugs that can lead to devastating acute and chronic clinical consequences in PWIDs and excess utilization of healthcare resources that could otherwise be prevented. Physicians should possess a high clinical suspicion for accurately diagnosing acute xylazine-induced naloxone-resistant overdoses and identifying chronic effects caused by xylazine, like ulcers and abscesses. More research is needed to study the trends of xylazine use across the US, its clinical effects, and the focus on therapy exclusively directed towards treating acute and chronic effects of xylazine. Extreme measures should be exercised by the local and federal government bodies to halt the increasing availability and use of Xylazine, along with other illicit drugs.
